# Application of Nonphosphorylative Metabolism as an Alternative for Utilization of Lignocellulosic Biomass

**DOI:** 10.3389/fmicb.2017.02310

**Published:** 2017-11-23

**Authors:** Maria K. McClintock, Jilong Wang, Kechun Zhang

**Affiliations:** Department of Chemical Engineering and Materials Science, University of Minnesota, Minneapolis, MN, United States

**Keywords:** nonphosphorylative metabolism, biochemicals, lignocellulose, pathway design, Metabolic Engineering

## Abstract

Production of chemicals via fermentation has been evolving over the past 30 years in search of economically viable systems. Thus far, there have been few industrially relevant chemicals that have seen commercialization, examples being lactic acid and ethanol. Currently, many of these fermentation processes still compete with food sources. In order to reduce this competition fermentation of alternative feedstocks, such as lignocellulosic biomass must to be utilized. Hemicellulosic sugars can be employed effectively for the production of chemicals by incorporating nonphosphorylative metabolism. This review covers nonphosphorylative metabolism, the pathways and enzymes involved, as well as the products that have been produced using nonphosphorylative metabolism.

Over the past 30 years the quest for bio-derived chemicals, both pharmaceutical and industrial products, has driven metabolic engineering and synthetic biology to identify and develop new pathways that are economically viable (Jambunathan and Zhang, 2014; Cheong et al., 2016). Challenges to economic viability frequently include: low titers; significant by-product production; the additional costs of inducers or antibiotics if pathways are not incorporated into the genome; and increased cost of edible biomass (Graham-Rowe, 2011; Burgard et al., 2016; Tai et al., 2016). One solution is the use of inedible or lignocellulosic biomass, which can be derived from sources such as agricultural waste. The United States alone can produce 1 billion dry tons from agriculture and this does not include the biomass that can be generated sustainably from forested land or the “waste” biomass generated from the logging industry (Graham-Rowe, 2011). Plant biomass is a composite material comprised of cellulose, hemicellulose, and lignin, with hemicellulose representing between 20 and 40% (Liao et al., 2016). Harsh acid/base or ionic liquid pretreatments are then used to free up the five and six carbon sugars within cellulose and hemicellulose for fermentation (Li et al., 2011). Utilization of all the sugars within lignocellulosic biomass is necessary to reduce competition with food resources and increase economic viability as well as renewability.

Several examples of industrially relevant chemicals derived from the tricarboxylic acid (TCA) cycle are 1,4-butanediol, glutamate, and succinic acid (Lee et al., 2003; Yim et al., 2011; Nishio et al., 2013). However, there are significant limitations to TCA cycle derivative production. The first limitation is low efficiency with respect to the utilization of pentose and hexose sugars (Liu et al., 2014). Furthermore, the longer pathway length due to glycolysis and PPP introduces more opportunities for by-products and bottlenecks in chemical production. This is evident in 1,4-butanediol production. Utilizing glycolysis the production pathway is composed of 21 steps, which greatly affects the production rate and yield (Yim et al., 2011; Tai et al., 2016).

Due to the limitations listed above industrial use of biological production has mostly been limited to production of antibiotics and food additives, however the past 10 years has seen a rapid development in fundamental technologies, as well as discovery of new enzymes. This development was necessary for complex pathway design and strain optimization (Gonzalez and Antoniewicz, 2017). For example, Yim et al. were able to use an algorithm to design a heterologous pathway for production of 1,4-butanediol (Yim et al., 2011). Furthermore, the sequencing of new species and characterization of new pathways and enzymes, which are available through open-source websites, such as KEGG, BLAST, and NCBI, gives researchers more possibilities for synthetic pathway design (Tai et al., 2016).

## Introduction to nonphosphorylative metabolism

Typically, metabolism in microbes occurs via glycolysis and the pentose phosphate pathways. Several pathways bypass the central metabolism steps, including: the Weimberg, the Dahms, and the Entner-Doudoroff pathways. The Weimberg pathway was originally described by Ralph Weimberg in a series of publications during the mid-twentieth century (Weimberg and Doudoroff, 1955; Weimberg, 1959, 1961). These pathways described the nonphosphorylative metabolism of D-ribose, D-xylose, and D- and L- arabinose in *Pseudomonas fragi* and L-arabinose in *Pseudomonas saccharophilia*. This pathway bypasses central metabolism to produce the TCA cycle intermediate 2-ketoglutarate (2KG) in less than six steps. Similarly, the Dahms pathway branches off from the Weimberg pathway at 2-keto-3-deoxy-D-xylonate and 2-keto-3-deoxy-L-arabonate to form pyruvate and glycoladehyde (Dahms and Anderson, 1969; Dahms, 1974). Finally, the Entner-Doudoroff bypasses glycolysis for the catabolism of glucose into glyceraldehyde phosphate and pyruvate (Gonzalez and Antoniewicz, 2017).

## Nonphosphorylative metabolism for arabinose

**Figure 1:**
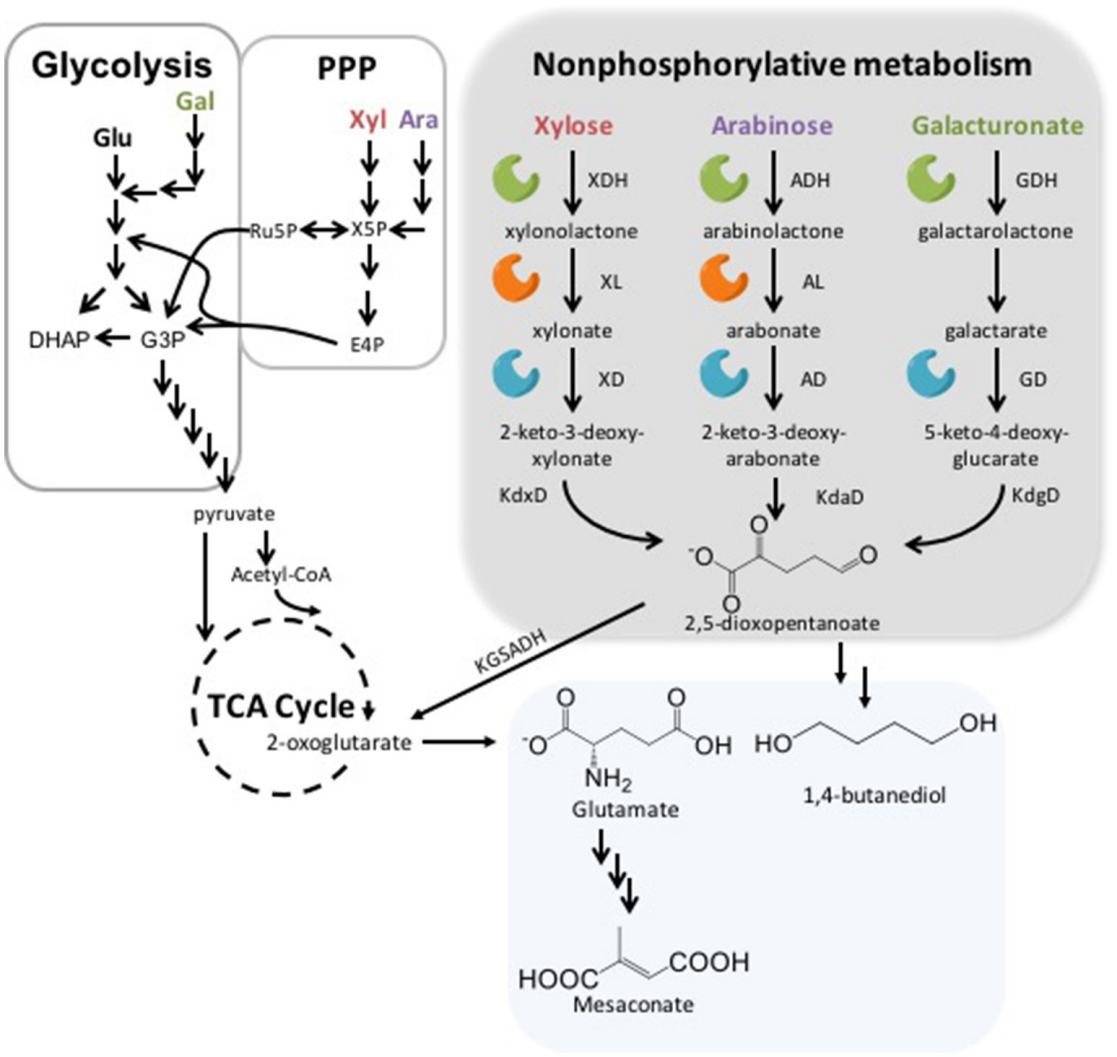
Glycolysis, the Pentose Phosphate Pathway (PPP) and Nonphosphorylative Pathways. In the gray box are the steps detailing degradation of xylose (Xyl), arabinose (Ara), and galacturonate (Gal) with the following abbreviations: xylose dehydrogenase (XDH), (XL), xylonate dehydratase (XD), 2-keto-3-deoxy-D-xylonate dehydratase (KdxD), arabinose dehydrogenase (ADH), arabinolactonase (AL), arabonate dehydratase (AD), 2-keto-3-deoxy-arabonate dehydratase (KdaD), galactronate dehydrogenase (GDH), D-galactarate dehydratase (GD) and 5-keto-4-deoxy-glucarate dehydratase (KdgD). 2-oxogluterate is then converted from 2,5-dioxopentanoate using ketoglutarate semialdehyde dehydrogenase (KGSADH). To the left of the gray box is an overview of Xyl, Ara, Gal, and glucose (Glu) assimilation using glycolysis and PPP. Finally, in the bottom box are some examples of products from nonphosphorylative metabolism.

Both L- and D- arabinose can be metabolized in a nonphosphorylative manner (Figure 1) (Weimberg, 1961; Stoolmiller and Abeles, 1966; Duncan, 1979). The first step in this pathway is conversion into L-arabinolactone using L-arabinose dehydrogenase (ADH). This is followed by L-arabinolactonase (AL) acting to covert L-arabinolactone into L-arabonate, which is then converted by L-arabonate dehydratase (AD) into 2-keto-3-deoxy-L-arabonate. 2-keto-3-deoxy-L-arabonate is metabolized to 2,5-dioxopentanoate via 2-keto-3-deoxy-L-arabonate dehydratase (KdaD). Finally, 2,5-dioxopentanoate is acted upon by 2-ketoglutarate semialdehyde dehydrogenase to make 2KG. These steps are the same for D-arabinose with the respective L- changed to D-.

Beyond *P. fragi* and *P. saccharophilia*, L-arabinose metabolism has been reported in several species of *Rhizobia* and *Burkholderia*, as well as *Azspirillum brasilense*, and *Herbaspirillum seropedicae* (Duncan, 1979; Novick and Tyler, 1982; Mathias et al., 1989; Moore et al., 2004; Watanabe et al., 2006; Tai et al., 2016). Full understanding of this pathway continues to develop as more pathways and additional research has provided several crystal structures and characterization of enzymes, including AD from *Rhizobium leguminosarum* and ADH from *Sulfolobus solfataricus* (Brouns et al., 2007; Rahman et al., 2017). In addition to being a useful tool for metabolic engineering, continued exploration of L-arabinose metabolism has led to new understanding of microbes. For example, L-arabinose metabolism has been linked to preventing virulence in species of *Burkholderia* (Moore et al., 2004). In another example, D-arabinose nonphosphorylative metabolism has been identified in several fungi including S*accharomyces cerevisiae* and *Candida albicans* where the final product is D-erythroascorbic acid (Kim et al., 1996, 1998).

## Nonphosphorylative metabolism for xylose

Efficient metabolism of xylose is critical to the use of lignocellulosic feedstocks, as it represents approximately one-third of the composition, making it a major target for fermentation use. Many bacterial and fungal species have the ability to utilize xylose for fermentation, but many of those that can use xylose well also give low product yields. This, however, has been greatly improved by metabolic engineering. Overall, there are three pathways for xylose metabolism, two of which produce D-xylulose, which is then phosphorylated into an intermediate in the pentose phosphate pathway (PPP). The third method is nonphosphorylative metabolism.

A number of species can use nonphosphorylative metabolism for D-xylose (Figure 1) assimilation, including *P. fragi, Burkholderia xenovorans*, and *Caulobacter crescentus* (Weimberg, 1961; Stephens et al., 2007; Tai et al., 2016). The first step in the conversion of D-xylose uses a xylose dehydrogenase (XDH) to catalyze the production of D-xylonolactone and NADH. D-xylonolactone is converted via D-xylonolactonase (XL) to D-xylonate, which is turned into 2-keto-3-deoxy-xylonate by a D-xylonate dehydratase (XD). 2-keto-3-deoxy-xylonate is then converted to 2,5-dioxopentanoate by means of 2-keto-3-deoxy-D-xylonate dehydratase (KdxD) before ultimately being converted into 2KG.

## Nonphosphorylative metabolism for galacturonate

Metabolism of galacturonate and other uronic acids occurs via three possible pathways: (1) isomerase pathway; (2) reductive pathway; and (3) oxidative pathway. The oxidative pathway resembles that of the xylose and arabinose nonphosphorylative pathways with the first step being the conversion of D-galacturonate into D-galactarolactone using uronate dehydrogenase (UDH) (Yoon et al., 2009; Pick et al., 2015). The subsequent step is then either D-galactarolactone spontaneously being hydrolyzed or catalyzed by lactonase to make D-galactarate (Andberg et al., 2012). Finally, D-galactarate is converted to 5-Keto-4-deoxy-D-glucarate by D-galactarate dehydratase (GD), which is subsequently converted into 2,5-dioxopentanoate by 4-deoxy-5-oxoglucarate hydrolase (KdgD) (Boer et al., 2010).

The nonphosphorylative metabolism pathway for galacturonate (Figure 1) is the least studied of the three pathways discussed in this review. UDH has been characterized in sizeable number of strains including: *Argobacterium tumefaciens, Fulvimarina pelagi, Streptomyces viridochromogenes, Oceanicola granulosus, and Pseudomonas syringae*. In 2012 a galactarolactone cycloisomerase from *Agrobacterium tumefaciens* capable of converting D-galactarolactone directly into 3-deoxy-2-keto-hexarate was characterized (Andberg et al., 2012). Finally, *Bacillus subtilis* was also seen to have an operon *ycbCDEFGHJ*, which may be responsible for the ability to utilize D-glucarate and D-galactarate as a sole carbon source (Hosoya et al., 2002). The authors believed that ycbC dehydrated 5-keto-4-deoxy-D-glucarate to 2,5-dioxopentanoate and this activity was confirmed in Tai et al. (2016).

## Chemicals derived from nonphosphorylative metabolism

### C5 compounds mesaconate/glutamate from nonphosphorylative metabolism

Nonphosphorylative metabolism promises high yield production of C5 compounds both theoretically and practically. Compared to the glycolysis pathway and the tricarboxylic acid (TCA) cycle, nonphosphorylative metabolism ensures high maximum theoretical yields, because xylose or arabinose flows to C5 compounds without excretion of CO_2_ (Weimberg, 1961; Stephens et al., 2007). High experimental yields have also been achieved when we employed nonphosphorylative metabolism to produce mesaconate and glutamate in *E. coli* (Bai et al., 2016). This is probably because in *E. coli*, nonphosphorylative metabolism is isolated from the endogenous metabolic pathways, and consequently, its intermediates cannot be competitively consumed by the intrinsic metabolic network but flow into the final products.

Mesaconate production was initially designed so that glycolysis pathway and tricarboxylic acid (TCA) cycle were applied to produce glutamate, and glutamate catabolic pathway from *Clostridium tetanomorphum* was introduced to convert glutamate into mesaconate (Wang and Zhang, 2015). Fermentation results showed that mesaconate yield was low (0.21 mol/mol glucose), indicating most of glucose has been consumed by competitive pathways. This wastes energy and generate by-products, such as acetate. A different mesaconate pathway was constructed so that nonphosphorylative metabolism converted xylose/arabinose into glutamate, and then mesaconate was produced by the same glutamate catabolic pathway from *C. tetanomorphum* (Bai et al., 2016). For the new engineered strains, glucose was specifically used to support bacterial growth and xylose/arabinose was used to produce mesaconate through nonphosphorylative metabolism. Fermentation results showed that mesaconate yield was more than 0.4 mol/mol xylose/arabinose without any optimization. Subsequent optimization strategies including screening nonphosphorylative operons with high activities, overexpression of xylose/arabinose transport system (*araE*), and deletion of *sucA*, ensure an even higher yield (0.85 mol/mol xylose/arabinose) (Bai et al., 2016). This work also suggested that the transport system is the rate-limiting step for nonphosphorylative metabolism-dependent mesaconate production.

Glutamate fermentation currently requires the combination of glycolysis pathway and TCA cycle. In order to improve glutamate production, different optimization strategies based on system biology, synthetic biology, and metabolic engineering, have been used over the last 60 years (Nunheimer et al., 1970; Asakura et al., 2007; Nishio et al., 2013). However, glutamate production yield was still low (0.68 mol/mol glucose) (Yoshimura et al., 1982; Delaunay et al., 1999; Nishio et al., 2013). Previous work also tried to use xylose or arabinose as feedstock to produce glutamate in engineered *Corynebacterium glutamicum* (Schneider et al., 2011; Meiswinkel et al., 2013). Similarly, glycolysis pathway and TCA cycle are still involved in this xylose or arabinose-derived glutamate production, and glutamate yield was very low (0.07 mol/mol arabinose) (Schneider et al., 2011). Recently, we employed nonphosphorylative metabolism to produce glutamate in *E. coli*, and modified the activity of the endogenous xylose catabolic pathway to mainly support bacterial growth and produce energy. The fermentation results showed after introducing nonphosphorylative metabolism, glutamate yield increased from 0.13 to 0.55 mol/mol xylose. Therefore, nonphosphorylative metabolism provides an ideal platform for C5 bio-chemicals production, because it has the potential to achieve high production yield even without complexed optimization steps.

### Production of 1,4-butanediol (BDO)

BDO is a significant commodity chemical that can be used as a building block for commercial products including plastic and elastic materials. Two studies were able to use nonphosphorylative metabolism or parts of the pathway for increased production of BDO from D-xylose (Liu and Lu, 2015; Tai et al., 2016). Production of BDO from the utilization of D-xylose, L-arabinose, or D-galacturonate reduces the number of steps for production from 21 to only six. BDO is formed when the conversion of 2,5-dioxopentanoate into butanedial by a 2-ketoacid decarboxylase is followed by an alcohol dehydrogenase. Each sugar was shown to be able to produce BDO in both shake flask fermentation and 1.3 L bioreactors with titers of 12 g/l for D-xylose, 15.6 g/l from L-arabinose, and 16.5 g/L from D-galacturonate. Further development of BDO production of the pathway was done by Wang et al. (2017) that included using an engineering protein for the conversion of the 1,2,4-butanetriol (BTO) by-product to BDO.

### Production of 3,4-dihydroxybutyric acid

Utilization of D-xylose via nonphosphorylative metabolism has also been incorporated into *E. coli* for the production of 3,4-dihydroxybutyric acid (3,4-DHBA) (Wang et al., 2017). 3,4-DHBA is the hydrolyzed form of 3-hydroxy-γ-butyrolactone (3HBL), a compound used in both pharmaceuticals and polymers. 3HBL is predominantly produced by chemical processes, but biological production of it and 3,4-DHBA would help to eliminate the use of harsh processes. The nonphosphorylative pathway for D-xylose metabolism was introduced until 2-keto-3-deoxy-D-xylonate. At this step keto acid decarboxylase is used to make 3,4-dihydroxybutanal, which can then be converted to the 3,4-DHBA. Fermentation of this produced 3,4-DHBA at a titer of 1.27 g/l with about 0.18 g/L of BTO produced.

## Conclusion

Nonphosphorylative metabolism while known since the mid-twentieth century has not seen widespread usage. The development of a selection platform for identification of new nonphosphorylative gene clusters provides the opportunity to expand the number of pathways available for utilization. Furthermore, many of these enzymes have not been subjected to protein engineering efforts or selective evolution leaving much to be expanded on in the future. The ability to use one of several of the hemicellulosic sugars is not only powerful because it expands feedstock options, but it also allows for the development of a system where glucose is predominantly used for cell growth while xylose, arabinose, and/or galacturonate are directly used for product production. The incorporation of nonphosphorylative metabolism is one way to increase the efficiency of sugar utilization, but these attributes need to be combined with strain engineering for increased resiliency to inhibitors present in lignocellulosic biomass hydrolysates. These changes will help to hasten the commercialization of fermentation products for the chemical industry.

## Author contributions

All authors listed have made a substantial, direct and intellectual contribution to the work, and approved it for publication.

### Conflict of interest statement

The authors declare that the research was conducted in the absence of any commercial or financial relationships that could be construed as a potential conflict of interest.
